# Novel *MYH7* Variant in the Neonate of a Mother with Gestational Diabetes Mellitus Showing Left Ventricular Hypertrophy and Noncompaction

**DOI:** 10.3390/genes15030381

**Published:** 2024-03-20

**Authors:** Sayaka W Ozawa, Satomi Inomata, Yukiko Hata, Shinya Takarada, Mako Okabe, Hideyuki Nakaoka, Keijiro Ibuki, Naoki Nishida, Fukiko Ichida, Keiichi Hirono

**Affiliations:** 1 Department of Pediatrics, Faculty of Medicine, University of Toyama, Toyama 930-0194, Japan; 2 Legal Medicine, Faculty of Medicine, University of Toyama, Toyama 930-0194, Japan; 3 Department of Pediatrics, International University of Health and Welfare, Tokyo 107-0052, Japan

**Keywords:** gestational diabetes mellitus, left ventricular noncompaction, *MYH7* gene

## Abstract

Background: Left ventricular hypertrophy (LVH) is a well-recognized cardiac dysfunction in infants of mothers with gestational diabetes mellitus (GDM). Left ventricular noncompaction (LVNC) is a cardiomyopathy that is morphologically characterized by numerous prominent trabeculations and deep intertrabecular recesses on cardiovascular imaging. However, there have been no case reports on neonates of mothers with GDM showing LVH and LVNC. Case presentation: A patient, with LVH of a mother with GDM, was delivered at 36 weeks of gestation. Prominent trabeculations in the LV, suggesting LVNC, instead of LVH, were apparent 1 week after birth. A heterozygous deletion variant in the *MYH7* gene (NM_000257.4: c.1090T>C, p.Phe364Leu) was discovered through genetic testing using a cardiomyopathy-associated gene panel in the patient and his father and the older brother who had LVNC. The patient is now 5 years old and does not have major cardiac events, although LVNC persisted. This is the first case of LVH secondary to a mother with GDM and LVNC with a novel variant in the *MYH7* gene. Conclusion: Genetic testing should be conducted to obtain an accurate outcome and medical care in a patient with LVH and subsequently prominent hypertrabeculation in the LV.

## 1. Introduction

The fetus of a pregnant woman with diabetes mellitus (DM) is at a high risk of teratogenicity and cardiac involvement due to left ventricular hypertrophy (LVH) [1,2,3]. LVH is a well-recognized cardiac dysfunction that occurs in the second to the third trimester in fetuses of mothers with gestational DM (GDM) [4,5,6]. The majority of those infants are clinically asymptomatic and experience LVH resolution within months after birth, most likely because they are no longer exposed to the aberrant intrauterine state.

Left ventricular noncompaction (LVNC) is a cardiomyopathy that is morphologically characterized by two layers of highly thickened myocardium, numerous prominent trabecular processes, and deep intertrabecular recesses connecting to the LV cavity [7,8,9]. With the development of next-generation sequencing (NGS) technologies, genetic variants have been reported to date in patients with LVNC [10]. Clinical symptoms are extremely diverse, ranging from infancy to adulthood onset, and from asymptomatic cases to severe cardiac dysfunction requiring consideration for heart transplantation. The onset of LVNC ranges from neonatal to infancy, school age, adolescence, and adulthood, depending on the case, and the clinical presentation is diverse, making it easily overlooked. Additionally, familial forms of LVNC are frequent, with various genetic patterns of inheritance observed. However, although LVNC was first reported clinically 30 years ago, LVNC and its pathogenesis are still poorly understood when compared to other cardiomyopathies, such as hypertrophic cardiomyopathy (HCM) and dilated cardiomyopathy (DCM).

However, no cases have been reported on neonates of mothers with GDM showing LVH and prominent hypertrabeculation in LV subsequently. Here, we present a neonate of a GDM mother with a novel variant in the *MYH7* gene exhibiting LVH and LVNC.

## 2. Case Presentation

A 39-year-old primigravida with a spontaneous singleton pregnancy (gravida 2 para 1) was managed with diet therapy because of gestational DM (GDM). Hemoglobin A1c was 5.8% at 35 weeks of gestation. On fetal echocardiography, no anomalies were observed during the gestation. The patient was a male infant who weighed 2.844 kg at birth and had Apgar scores of 4 and 5 at 1 and 5 min, respectively. Resuscitation, including artificial ventilation, was required because of weak breathing and bradycardia at birth. Tachypnea persisted despite the establishment of spontaneous breathing upon transfer to the neonatal intensive care unit. Upon admission, saturated oxygen was 96%, and venous blood gas analysis was almost normal except for hypercarbia: pH of 7.306, partial pressure of carbon dioxide of 55.3 mmHg, bicarbonate of 26.8 mEq/L, and base excess of −0.7 mEq/L on room air. The levels of liver transaminase and creatine kinase were not elevated, and the inflammatory response was negative. X-ray showed cardiomegaly (65% of cardiothoracic ratio) and pulmonary congestion.

**Figure 1:**
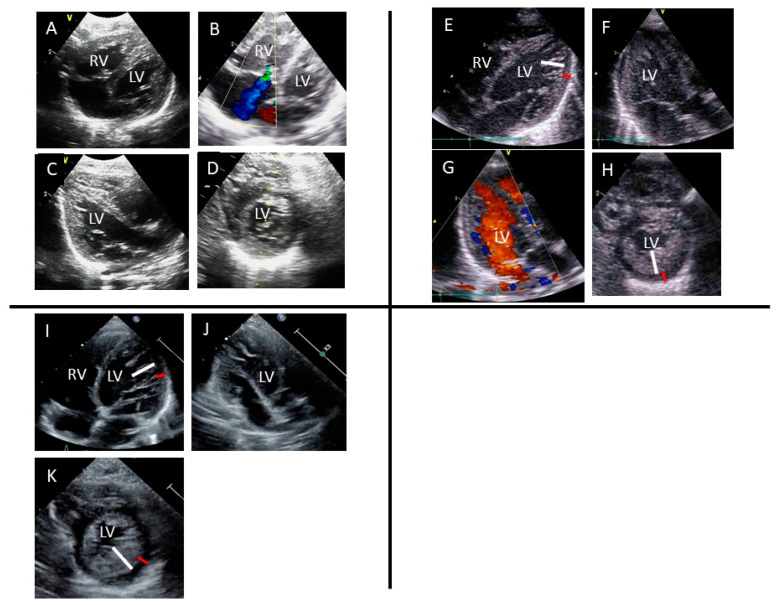
An ultrasound image showing an abnormal, highly trabeculated left ventricular myocardium (**A**–**K**): four-chamber (**A**,**B**), long-axis (**C**), and short-axis (**D**) views showing LVH at birth. B demonstrates moderate tricuspid regurgitation (blue flow) due to pulmonary hypertension; four-chamber (**E**), long-axis (**F**,**G**), and short-axis (**H**) views 7 days after birth. E and H demonstrate compacted layer (red line) and noncompacted layer (white line). Noncompacted layer shows deep intertrabecular recesses connecting to the LV cavity. G demonstrate red blood flow enter into deep intertrabecular recesses; four-chamber (**I**), long-axis (**J**), and short-axis (**K**) views at 1 year old. Similarly, I and K demonstrate compacted layer (red line) and noncompacted layer (white line). LV, left ventricle; RV, right ventricle.

Furthermore, echocardiography revealed a normal LV ejection fraction (56%) and LVH (the Z-scores of interventricular septum and LV posterior wall thickness were 1.03 and 2.60, respectively), moderate tricuspid regurgitation, and pulmonary hypertension (Figure 1). Other extracardiac malformations were not observed. The patient was diagnosed with pulmonary hypertension and tricuspid valve regurgitation secondary to neonatal asphyxia and LVH secondary to GDM. Oxygenation was performed, which improved breathing. However, oral nutrition was inadequate, and heart failure was suspected. Although pulmonary hypertension and tricuspid regurgitation had improved 1 week after delivery, substantial trabeculations in the LV were noticeable instead of LVH, suggesting LVNC with reduced LV contraction (LVEF of 41%) (Figure 1). The patient’s symptoms gradually improved, and he was discharged 22 days after birth. LVNC still persisted at 1 year old (Figure 1). 

After obtaining informed consent from the parents of the patients, DNA was isolated from whole blood samples. NGS of 182 cardiac disease-related genes associated with cardiomyopathy and channelopathies was performed using the Ion PGM System (Life Technologies, Carlsbad, CA, USA) [10]. Only variants present in genes other than the top 1% of genes with high variability and meeting acceptable quality scores (Phred-scaled genotype quality score, ≥20; read depth, ≥15; and Allele Fraction, ≥35) were included. Following quality control procedures, a total of 303,993 variants were obtained from the dataset. These variants were utilized for downstream analysis. Sanger sequencing was used to validate the results of NGS for all pathogenic variant candidates that passed these selection criteria. For this purpose, the nucleotide sequences of the amplified fragments were directly sequenced bidirectionally using the BigDye Terminator v3.1 Cycle Sequencing Kit (Applied Biosystems, Foster City, CA, USA), and sequence analysis was conducted using the ABI 3130xl automated sequencer (Applied Biosystems).

We conducted primary, secondary, and tertiary analysis, including optimization of signal processing, base calling, sequence alignment, and variant analysis, using Torrent Suite and Ion Reporter Software 5.0 (Life Technologies). The allele frequencies of all detected variants were determined using the gnomAD (v2.1.1) database, which includes data from 1208 Japanese individuals, and the Japanese Multi Omics Reference Panel (jMorp) ToMMo 54KJPN. Variants with a minor allele frequency of 0.005 or higher in the gnomAD and HGVD populations were excluded. Variant evaluation was manually performed based on detailed information obtained from ClinVar (https://www.ncbi.nlm.nih.gov/clinvar/, accessed on 11 March 2024) and the Human Gene Mutation Database (HGMD, http://www.hgmd.cf.ac.uk/ac/index.php, accessed on 11 March 2024). Variants were classified according to the American College of Medical Genetics and Genomics (ACMG) guidelines. To assess the pathogenicity of the remaining variants, we utilized five different in silico prediction algorithms: SIFT, Align GVGD, MutationTaster2, PolyPhen-2, and CADD. Variants predicted to be deleterious or pathogenic by at least four of the five in silico algorithms were considered to have a high likelihood of pathogenicity (Table 1).

**Figure 2:**
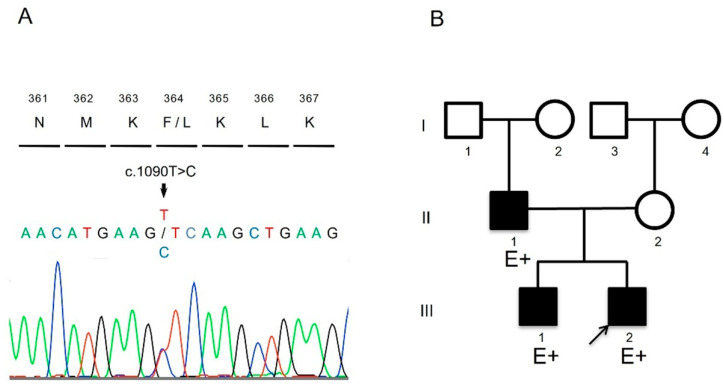
(**A**) The results of Sanger sequencing of target alleles (A: green, C: blue, T: red, G: black). (**B**) Family pedigree. E+ indicates variant positivity. Black color indicates that the patient is affected by LVNC.

The patient harbored a novel heterozygous missense mutation (NM_000257.4:c.1090T>C, p.Phe364Leu) in the *MYH7* gene (Figure 2). This mutation has not been previously reported in public databases of the general population, or is extremely rare, and it was predicted to be deleterious by multiple bioinformatics prediction tools (Table 1). ClinVar classified it as uncertain significance. According to the guidelines of the American College of Medical Genetics and Genomics and the Association for Molecular Pathology, the pathogenicity of this variant was considered likely pathogenic. It has been previously reported that the amino acid change Met362Arg in *MYH7* includes all elements necessary for actin to move relative to myosin during adenosine triphosphate hydrolysis [11]. The patient’s father and brother also harbored the same mutation, and mild left ventricular noncompaction (LVNC) was demonstrated in them through echocardiography (Figure 2 and Table 2). For these reasons, we considered this novel mutation to be the cause of the patient’s condition.

The patient’s father and older brother had the same variant, and echocardiography showed that they had mild LVNC (Figure 2 and Table 2). 

The patient is now 5 years old; he has experienced no major cardiac event such as hospitalization for heart failure and cardiac arrest, although LVNC persists.

## 3. Discussion

To the best of our knowledge, this is the first pediatric case of a mother with GDM who showed LVH and developed into LVNC caused by a novel variant in the *MYH7* gene. LVH has a spontaneous resolution within months in most of the neonates of mothers with GDM. However, in our patient, prominent trabeculations in the LV, instead of LVH, were apparent 1 week after birth, suggesting LVNC.

GDM develops during the latter half of pregnancy and is associated with fetal macrosomia, cardiomyopathy, and increased incidence of perinatal complications and mortality [12]. Typically, LVH spontaneously resolves within months in most neonates of mothers with GDM. However, in our patient, prominent trabeculations in the LV, instead of LVH, were evident 1 week after birth, indicating LVNC. A previous study reported that LVH develops from the late second to the third trimester in the fetuses of mothers (29–35 weeks of gestation) with DM [13]. The perinatal condition of GDM may affect LVH, which improves a few months after birth, with no further exposure to the aberrant intrauterine condition. 

In LVNC, it is believed that this process of compaction is arrested, leaving behind a spongy fetal myocardium, while, conversely, compacted myocardium with reduced formation persists [14,15]. During the developmental process of the mammalian heart, the ventricles undergo a series of morphogenetic events, including trabecularization and compaction. Recent studies in mouse models have elucidated the importance of temporal and spatial coordination of developmental gene networks, primarily centered around transcription factors, in trabeculation. During this stage, cardiomyocytes need to interact with their surrounding environment, including the endocardium, epicardium, extracellular matrix, other cardiomyocyte factors, and epigenetic factors. Trabecular formation necessitates interaction between the endocardium and myocardium, with this interaction in late development contributing to ventricular compaction. These endocardial–myocardial pathways are mainly driven by signals from the neuregulin1/ErbB, Notch, and BMP10 pathways. The Notch1 and neuregulin1 pathways dynamically interact to regulate extracellular matrix dynamics and trabecular growth, establishing the basis of trabecular architecture. The Dll4-Notch1 pathway, Mib1 pathway, BMP10 pathway, and TGF-β pathway regulate trabecular compaction. The Hey2 or Nppa systems are implicated in Dll4-Notch1 variations. Nrg1, Bmp10, and Hey2 have been reported to be Notch1-dependent. Recent studies have shown that Dll4-Notch1 signaling induces trabecular formation and coordinates its patterning and compaction with coronary formation to form mature chambers. Human germline variants in MIB1, encoding an E3 ubiquitin ligase that facilitates endocytosis of Notch ligands Delta and Jagged, lead to LVNC, with affected individuals exhibiting reduced Notch1 activity and targets. Kodo et al. demonstrated that Tbx20 regulates the expression of TGF-β signaling regulators, including Prdm16, an SMAD2/3 inhibitor of TGF-β signaling, through binding [11] Downregulation of Erbb2 signaling in mature trabeculae requires the endocytosis-adaptive proteins Numb and Numbl.

The genetic background of LVNC includes both sporadic and familial cases. A significant proportion (28%) of familial cases exhibits genetic heterogeneity, with suspected inheritance patterns including autosomal dominant, autosomal recessive, mitochondrial gene abnormalities, and potential X-linked inheritance. Approximately 40% are believed to have specific genetic mutations. Van Waning et al. conducted a systematic review, identifying a total of 80 genes reported as genetic causes of LVNC [16]. These genes were classified based on molecular function into sarcomere genes, arrhythmia genes, non-sarcomere genes, non-arrhythmic cardiomyopathy genes, X-linked genes, CHD-related genes, and mitochondrial dysfunction genes. The majority of genetic causes were single mutations (85%), with missense mutations in autosomal dominant inheritance being the most common (55%). X-linked gene abnormalities accounted for 7% of patients, with *TAZ* being the most prevalent (6%). Chromosomal abnormalities were present in 6% of patients, with 88% diagnosed in childhood. Over 50% of gene abnormalities were found in sarcomere genes, with *MYH7* being the most common (25%). Mutations in arrhythmia genes were observed in 11%, with *HCN4* being particularly prevalent (4%).

Variants in *MYH7* appear to be a significant cause of LVNC, accounting for almost half of the pathogenic variants identified in the Japanese pediatric population [10]. The Met362Arg amino acid changes in *MYH7*, which we previously reported to contain all the necessary elements to generate movement of actin relative to myosin during adenosine triphosphate hydrolysis [11]. The present variant has not yet been published in public databases, but it was detected in the affected family members and was well segregated. Thus, the variant in *MYH7* detected in the present study may affect functional and structural domains. In mouse models, many genes associated with myocardial cell proliferation result in LVNC-like phenotypes, indicating a broad impact that requires tissue-specific genetic analysis. Cell-type-specific deletion of numerous genes studied in mice was necessary to elucidate their roles in proliferation, trabecular formation, and compaction. Since the products of these genes function in other cells as well, gene deletions are expected to result in embryonic lethality. In contrast, many of the variants associated with human LVNC involve sarcomere genes, which are part of the muscle fiber structure and are associated with contractile function. These genes are also known to be involved in other inherited cardiomyopathies. Thus, the variant in the *MYH7* gene in this case is considered typical among the genes associated with human LVNC and given that this gene is reported to be involved in numerous other inherited cardiomyopathies, it is conceivable that they also exert significant influence in this case.

In the present case, LVNC was discovered 1 week after birth, whereas LVH was discovered at birth. This could be attributed to the abnormal intrauterine conditions in GDM, specifically insulin-like growth factor 1 (IGF-1)-mediated cardiomyocyte growth and proliferation [17,18]. Fetal hyperinsulinemia and IGF-1, which promotes cardiac myocyte proliferation and hypertrophy, are thought to be the causes of LVH. Our earlier research demonstrated that patients with variants in the *MYH7* gene were frequently diagnosed with heart failure at 6 months of age, which was the average age of LVNC onset [10]. In this instance, LVNC was discovered 1 week after birth, whereas LVH was discovered at birth. This is presumably attributed to exposure to the abnormal intrauterine condition of GDM, specifically IGF-I-mediated cardiomyocyte growth and proliferation. Recently, we published a case of a neonate who developed complications after surgery and who had variants in the *MYH7* and *CD36* genes [19]. Fatty acid translocase, usually referred to as CD36, is a transmembrane glycoprotein belonging to the class B scavenger receptor family. Defective CD36 is a likely candidate gene for impaired fatty acid metabolism, glucose intolerance, and diabetes. In the presence of unfavorable circumstances, some pediatric patients with CD36 deficiency were susceptible to myocardial damage. Thus, our previous case is consistent with our theory that LVNC may be caused by a complicated interaction between genetics and metabolic condition. 

Only one report has been made on LVNC and diabetic embryopathy. The autopsy results of a late-preterm female baby with biventricular noncompaction and diabetic embryopathy were reported [20]. However, this report was that of a patient with multiple congenital malformations, cardiac defects, and central nervous system disorders, indicating diabetic embryopathy and that the patient’s mother had been complicated by pregestational DM before pregnancy. Moreover, the genetic status was not evaluated. Contrarily, our case could highlight the genetic background of LVNC related to the influence of the perinatal condition of GDM.

Interestingly, the brother and father were asymptomatic and LVNC was detected during family screening when the brother was diagnosed with LVNC, whereas the patient’s LVNC became apparent after LVH in neonate. The difference between the patient and his brother was that the mother did not have GDM in the brother’s case, whereas the brother did, suggesting that GDM may have accentuated LVNC and its symptoms. LVNC presents with several distinct morphologies owing to the nature of associated phenotypes. Given such diversity in etiology, it is not surprising that the genetic nature of LVNC is heterogeneous. The progression of GDM and LVNC observed in this case may challenge the conventional and a priori interpretation of non-compaction forms as genetic diseases or cardiomyopathies.

## 4. Conclusions

To the best of our knowledge, this is the first case of a mother with GDM who showed LVH and developed into LVNC with a novel variant in the *MYH7* gene. Although LVNC is a lifelong disease that is highly heterogeneous in both phenotype and genotype, genetic testing should be conducted to obtain an accurate outcome and medical care in a patient with LVH and subsequently prominent hypertrabeculation in the LV.

## Figures and Tables

**Table 1:** Variants identified in the patient.

Gene	Accession ID	Protein	cDNA	dbSNP	gnomAD	ToMMo 54KJPN	SIFT	polyphen2	GVGD	Mutation Taster	CADD	Clin Var	ACMG Classification
*MYH7*	NM _000257.4	p.Phe364Leu	c.1090T>C	rs1793561613	n/a	0.000009	Disease causing	0.156	C15	Disease causing	23.8	Uncertain significance	Likely pathogenic; PM1, PM2, PP3, PP4

**Table 2:** The differences in phenotypes in the individuals with the *MYH7* variant.

	Sex	Age at Diagnosis(y)	Symptom	Cardiomegaly	Reduced Ejection Fraction	Area of LV Noncompaction
Proband	male	0	Heart failure	yes	yes	AW, LW, PW, SW and apex
Brother	male	2	no	no	no	LW, PW and apex
Father	male	40	no	no	no	PW and apex

AW; anterior wall, LW; lateral wall, PW; posterior wall, SW; septal wall.

## Data Availability

The authors confirm that the data supporting the findings of this study are available within the article.
